# Supplementary material for: Indane Based Molecular Motors: UV-Switching Increases Number of Isomers
Source: Molecules. 2022 Oct 9;27(19):6716. doi: 10.3390/molecules27196716 (PMC9570826; doi:10.3390/molecules27196716)

Supplementary materials

Indane based molecular motors. UV-switching increases number of isomers.

Valeriy P. Shendrikov 1,2, Anna S. Alekseeva 1, Erik F. Kot 1, Konstantin S. Mineev 1, Daria S. Tretiakova 1, Abdulilah Ece 3 and Ivan A. Boldyrev 1,*

1 Shemyakin-Ovchinnikov Institute of Bioorganic, Chemistry of the Russian Academy of Sciences, GSP-7, Ulitsa Miklukho-Maklaya, 16/10, 117997 Moscow, Russia
2 Higher Chemical College, Mendeleev University of Chemical Technology of Russia, Miusskaya sq. 9, 125047 Moscow, Russia
3 Department of Pharmaceutical Chemistry, Faculty of Pharmacy, Biruni University, Istanbul 34010, Turkey
* Correspondence: ivan@lipids.ibch.ru; Tel.: +7-495-330-66-10

1. NMR spectra of target and intermediate compounds 2

2. Energy barriers of rotation 6

3. Molecular orbitals 8

Section S1.
NMR of target and intermediate compounds

Chemical shifts of 4-aphin, 6-aphin

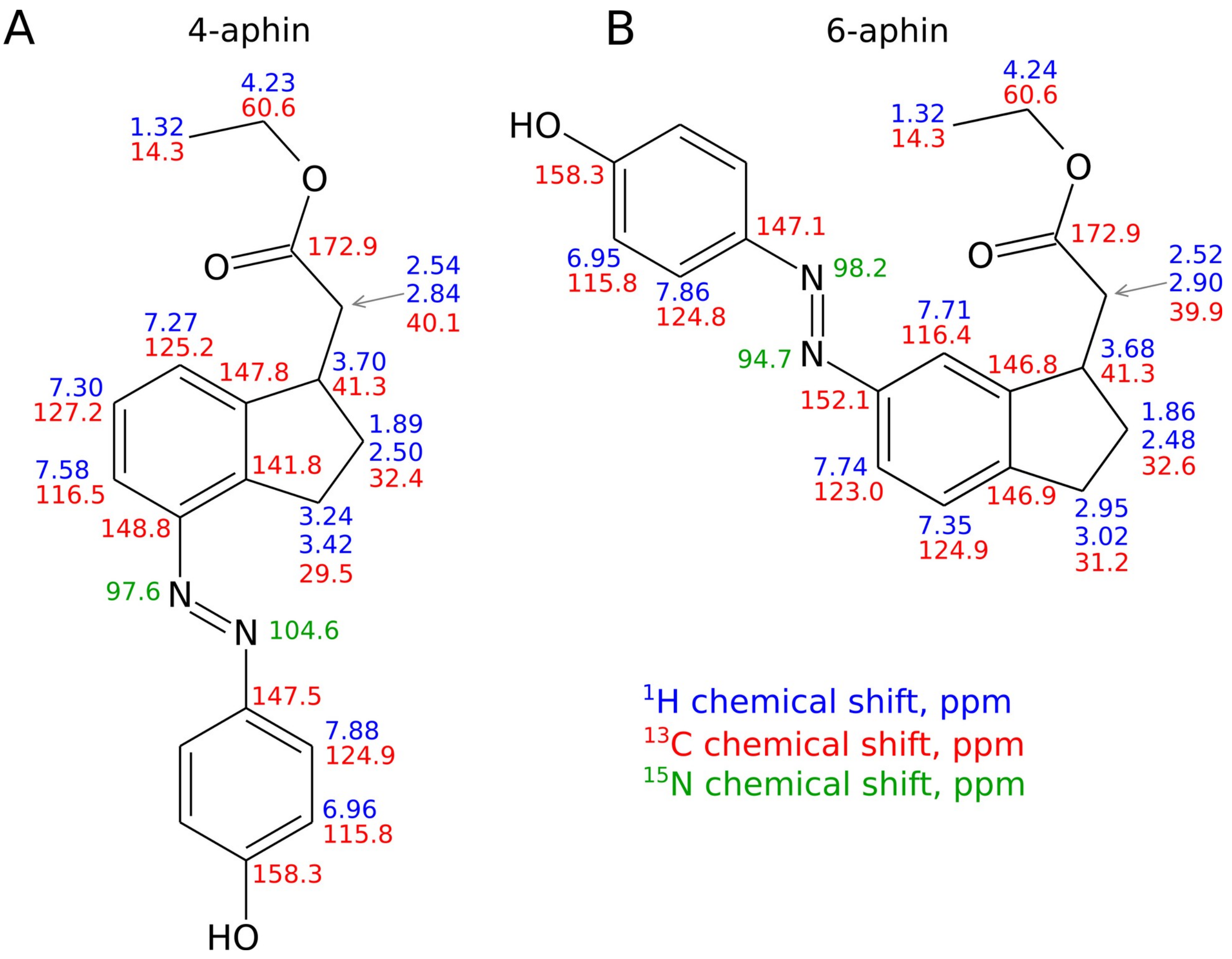

Chemical shifts of 4-aphin, 6-aphin

6-aphin

ethyl 2-{6-[(1E)-2-(4-hydroxyphenyl)diazen-1-yl]-2,3-dihydro-1H-inden-1-yl}acetate

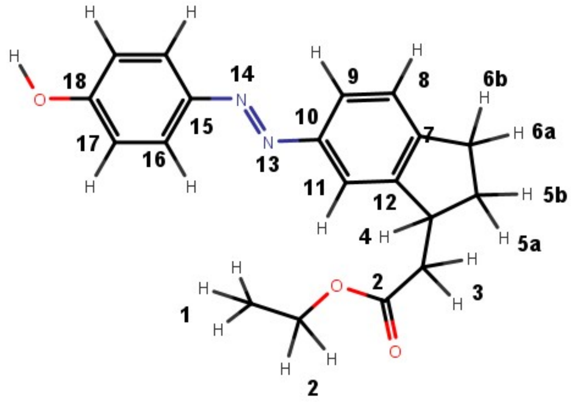

4-aphin

ethyl 2-{4-[(1E)-2-(4-hydroxyphenyl)diazen-1-yl]-2,3-dihydro-1H-inden-1-yl}acetate

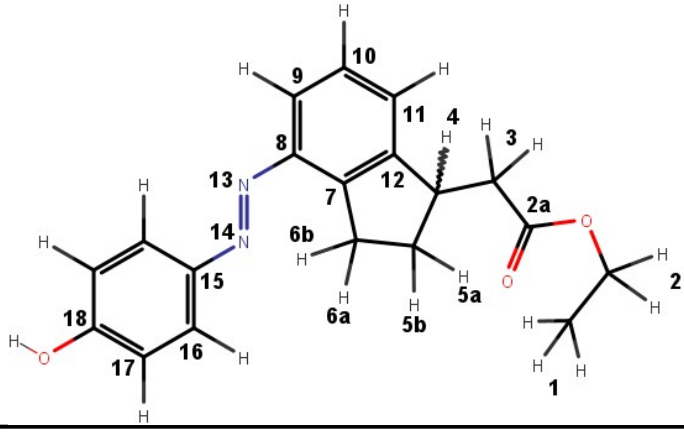

Atom #	^{1}H	^{13}C	^{15}N	^{1}H	^{13}C	^{15}N
**1**	1.32 t	14.3	-	1.32	14.3	-
**2**	4.24 m	60.6	-	4.23	60.6	-
**2a**	-	172.9	-	-	172.9	-
**3**	a: 2.52 m<br>b: 2.90 m	39.9	-	2.54<br>2.84	40.1	-
**4**	3.68	41.3	-	3.70	41.3	-
**5**	a: 1.86<br>b: 2.48	32.6	-	1.89<br>2.50	32.4	-
**6**	a: 2.95<br>b: 3.02	31.2	-	3.24<br>3.42	29.5	-
**7**	-	146.9	-	-	141.8	-
**8**	7.35	124.9	-	-	148.8	-
**9**	7.74	123.0	-	7.58	116.5	-
**10**	-	152.1	-	7.30	127.2	-
**11**	7.71	116.4	-	7.27	125.2	-
**12**	-	146.8	-	-	147.8	-
**13**	-	-	94.7	-	-	97.6
**14**	-	-	98.2	-	-	104.6
**15**	-	147.1	-	-	147.5	-
**16**	7.86	124.8	-	7.88	124.9	-
**17**	6.95	115.8	-	6.96	115.8	-
**18**	-	158.3	-	-	158.3	-

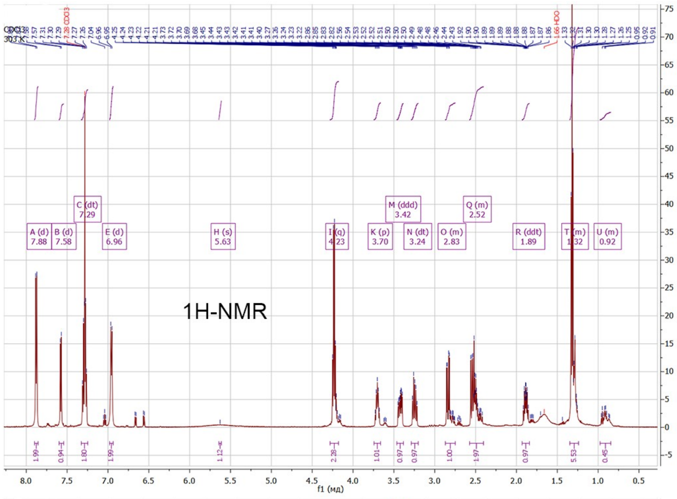
1H-NMR
f1 (мд)

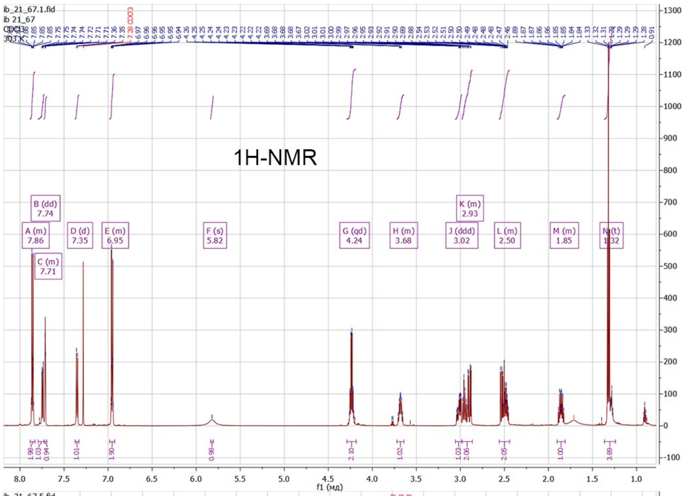
ib_21_67.1.fid
ib 21_67
1H-NMR
f1 (мд)

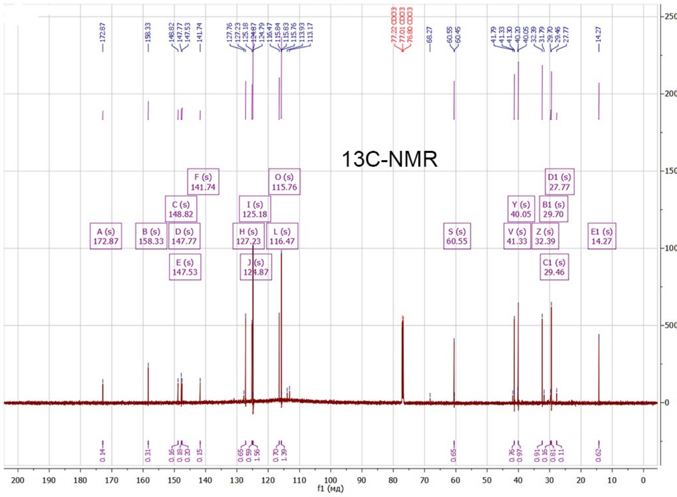
13C-NMR
f1 (мд)

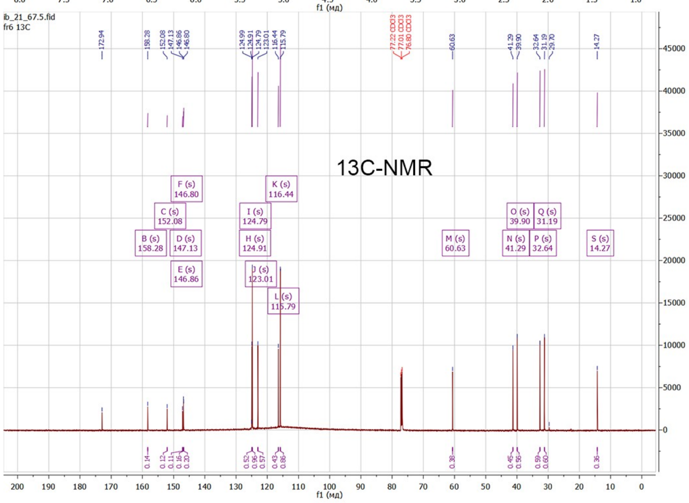
ib_21_67.5.fid
fr6 13C
13C-NMR
f1 (мд)

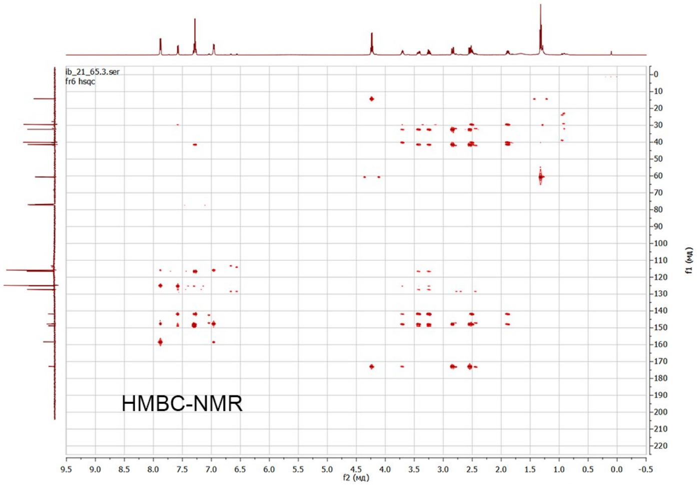
ib_21_65.3.ser
fr6 hsqc
HMBC-NMR
f1 (мд)
f2 (мд)

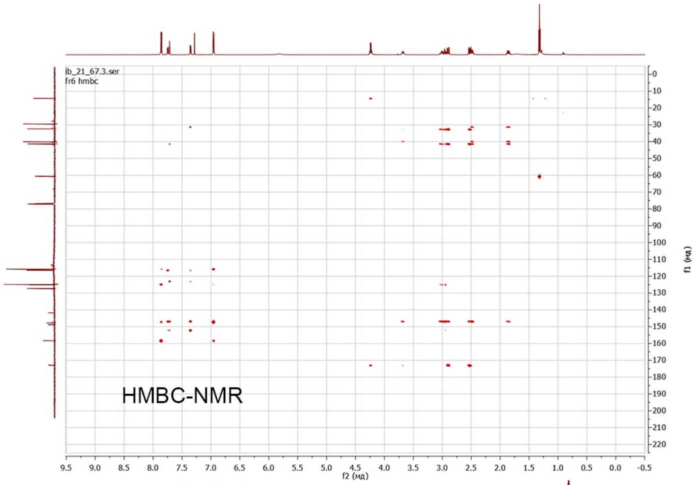
ib_21_67.3.ser
fr6 hmbc
HMBC-NMR
f1 (мд)
f2 (мд)

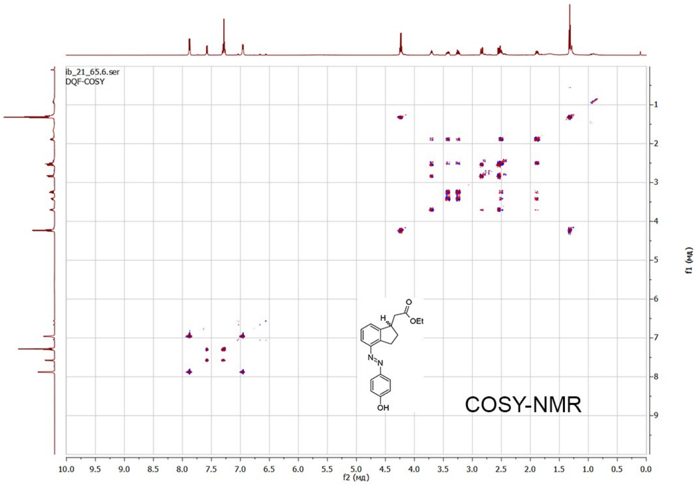
ib_21_65.6.ser
DQF-COSY
OEt
OH
COSY-NMR
f1 (мд)
f2 (мд)

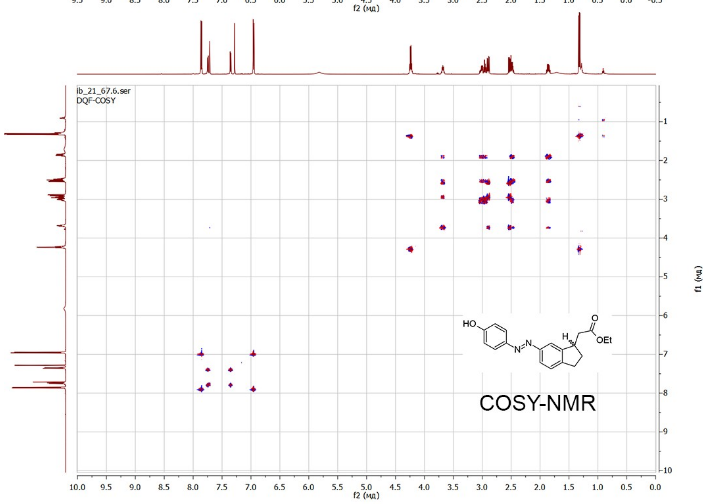
ib_21_67.6.ser
DQF-COSY
HO
OEt
COSY-NMR
f1 (мд)
f2 (мд)

NMR spectra of intermediate compounds

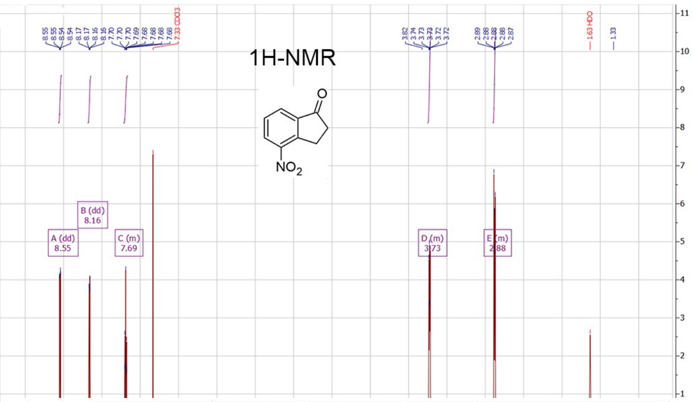

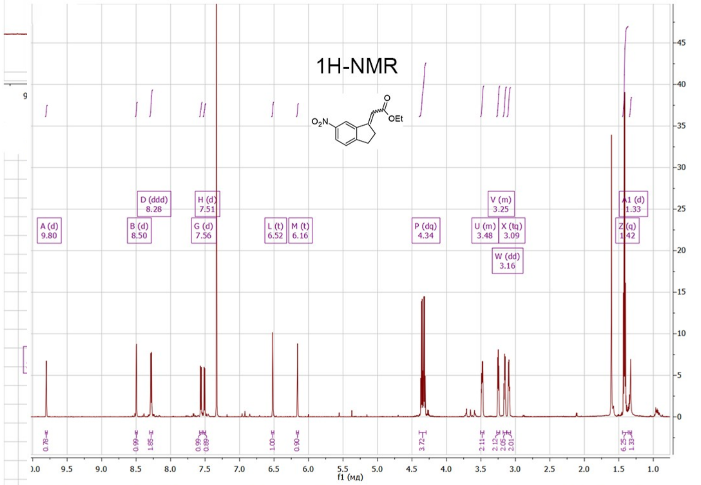

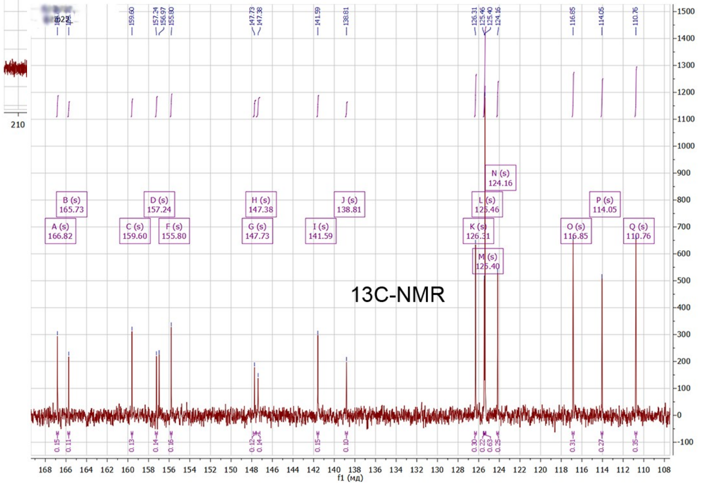

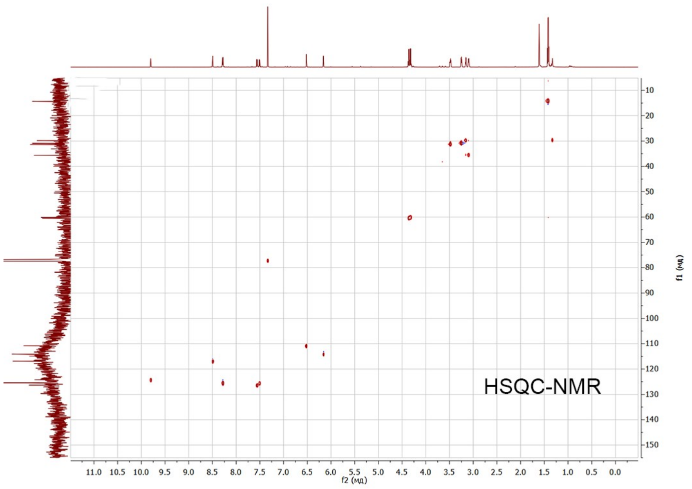

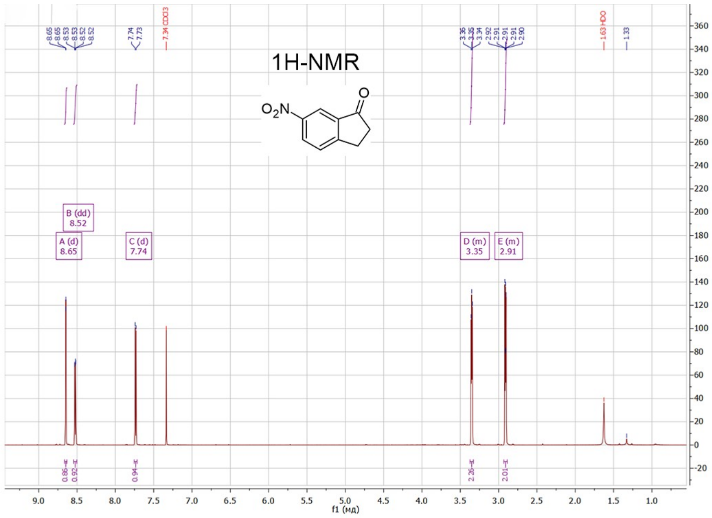

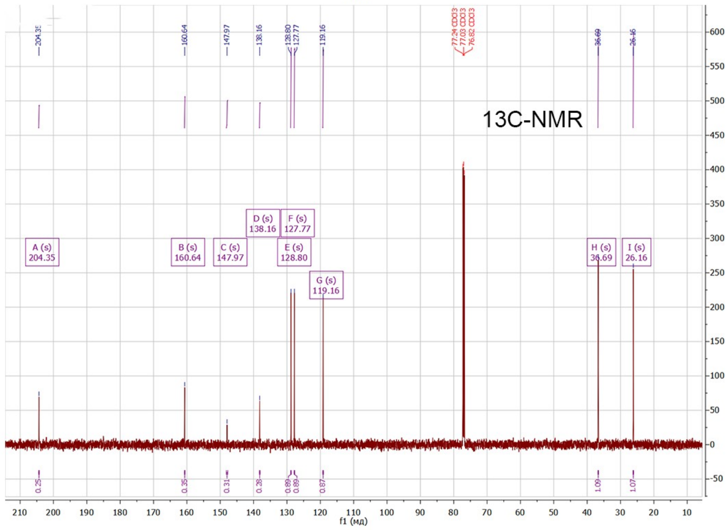

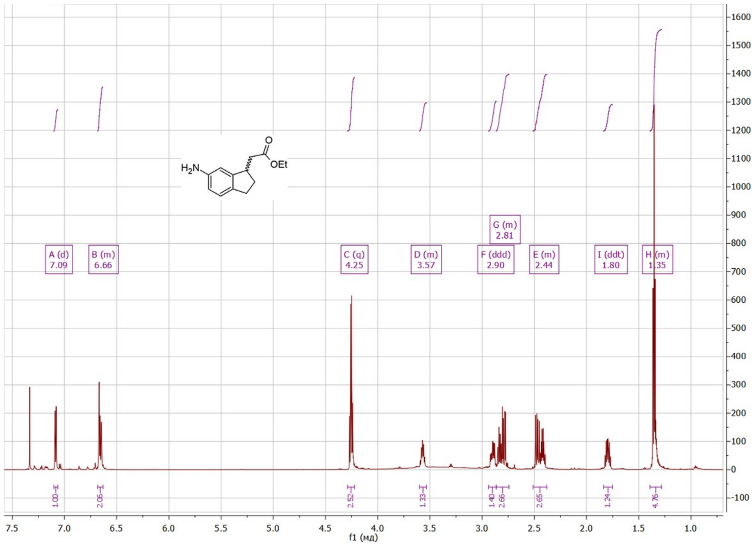

Section S2. Energy barriers of rotation.

Energy barriers of rotation calculated at BP86 level of theory

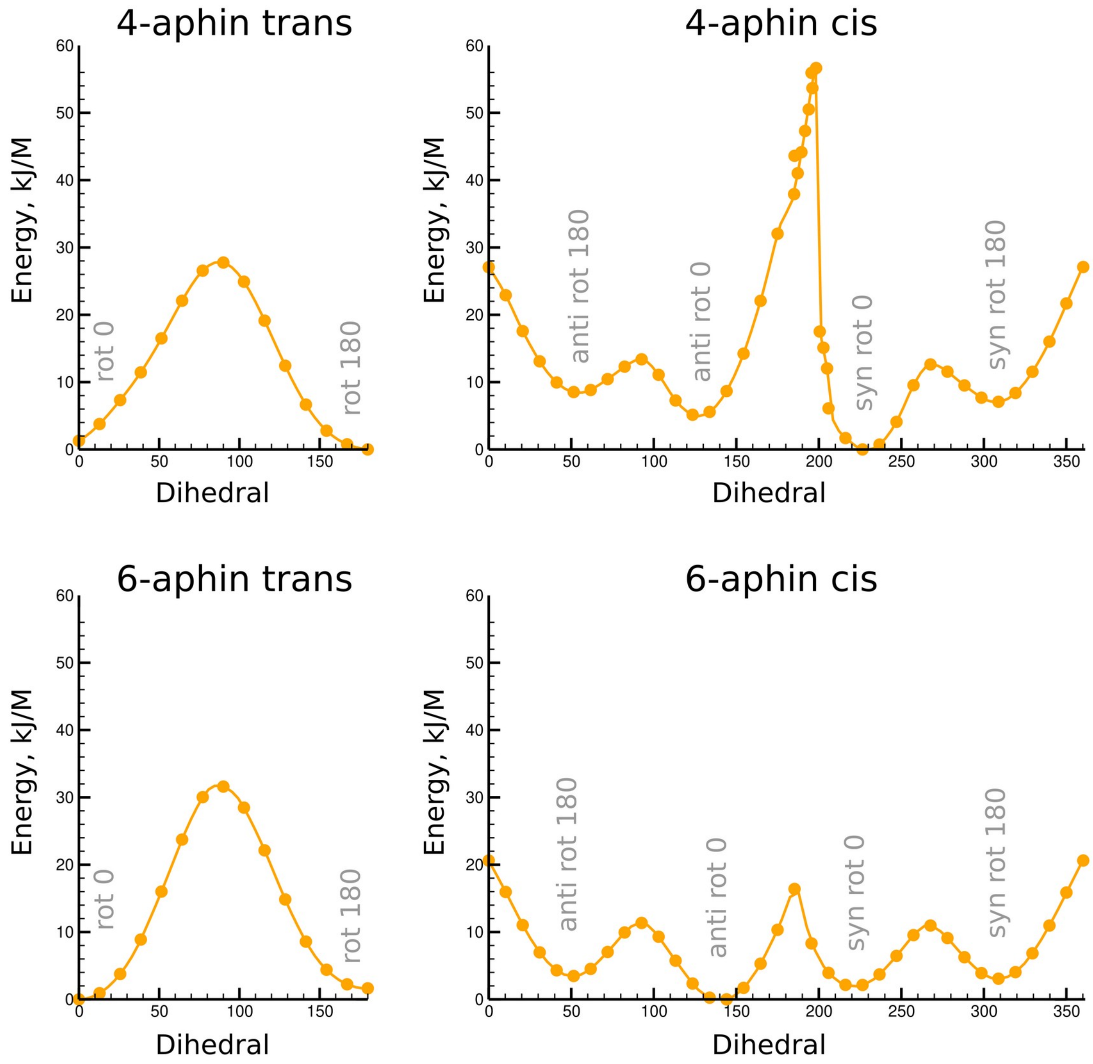

Extremum points of the above calculated at B3LYP level of theory

4-aphin

**Dihedral**	51,47	92,65	123,53	195,59	226,47	267,65	308,82
**E, H**	-1071,09712	-1071,08504	-1071,08758	-1071,06496	-1071,08847	-1071,08538	-1071,08749
**E, kJ/mol**	3,54	9,01	2,31	61,72	0	8,1	2,57

6-aphin

Dihedral	51,47	92,65	144,12	185,29	226,47	267,65	308,82
E, H	-1071,08660	-1071,08359	-1071,08694	-1071,08053	-1071,08677	-1071,08336	-1071,08650
E, kJ/mol	0,9	8,81	0	16,85	0,46	9,4	1,16

B3LYP vs BP86 at stationary point

4-aphin

	trans_rot180	trans_rot0	cis_anti_rot180	cis_anti_rot0	cis_syn_rot180	cis_syn_rot0
E, H (B3LYP)	-1071,112446	-1071,112043	-1071,095227	-1071,095513	-1071,095531	-1071,096916
E, kJ/mol (B3LYP)	0,0	1,1	45,2	44,5	44,4	40,8
E, H (BP86)	-1070,552879	-1070,552389	-1070,523108	-1070,524394	-1070,523660	-1070,526357
E, kJ/mol (BP86)	0,0	1,3	78,2	74,8	76,7	69,6

6-aphin

	trans_rot180	trans_rot0	cis_anti_rot180	cis_anti_rot0	cis_syn_rot180	cis_syn_rot0
E, H (B3LYP)	-1071,113069	-1071,113641	-1071,094469	-1071,094707	-1071,094533	-1071,095148
E, kJ/mol (B3LYP)	1,5	0,0	50,3	49,7	50,2	48,6
E, H (BP86)	-1070,552181	-1070,552803	-1070,521928	-1070,522286	-1070,521776	-1070,523098
E, kJ/mol (BP86)	1,6	0,0	81,1	80,1	81,5	78,0

Section S3.

Frontier molecular orbitals calculated at B3LYP level of theory.

4-aphin R trans rot 180

ΔE = 3.6058 eV, 343.8 nm

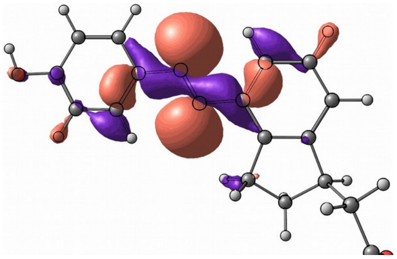

HOMO-1

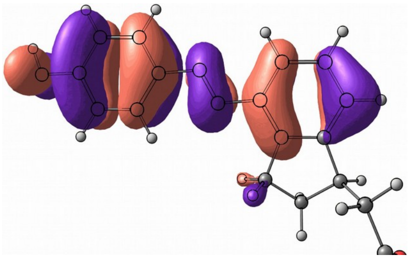

HOMO

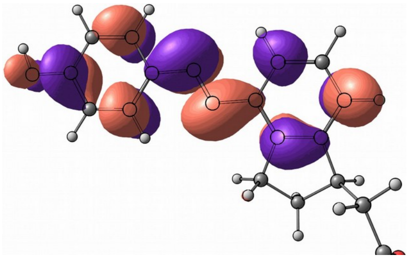

LUMO

4-aphin R trans rot 0

ΔE = 3.7051 eV, 334.6 nm

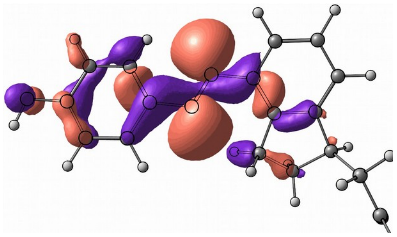

HOMO-1

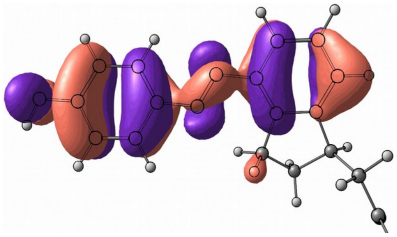

HOMO

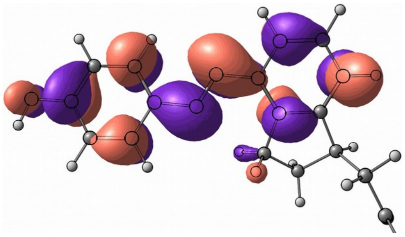

LUMO

4-aphin cis syn rot 180

ΔE = 3.6989 eV, 335.2 nm

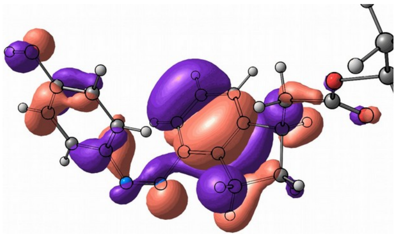

HOMO-1

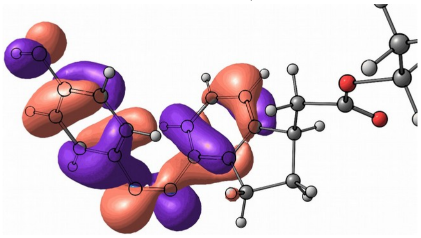

HOMO

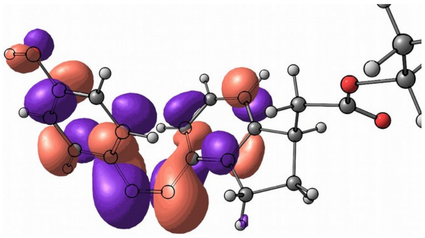

LUMO

4-aphin cis syn rot 0

ΔE = 3.7187 eV; 333.4 nm

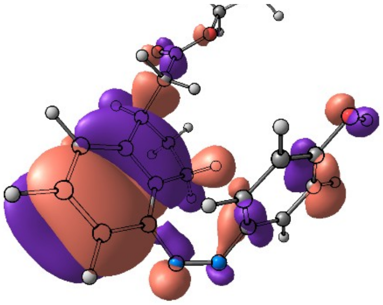

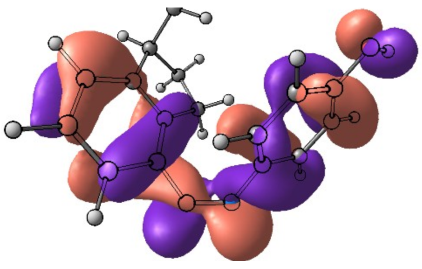

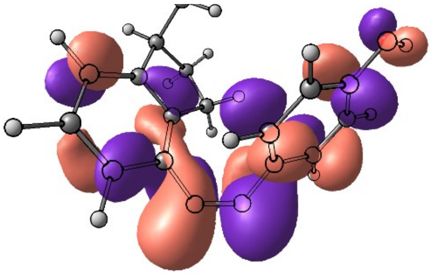

4-aphin cis anti rot 180

ΔE = 3.6934 eV; 335.7 nm

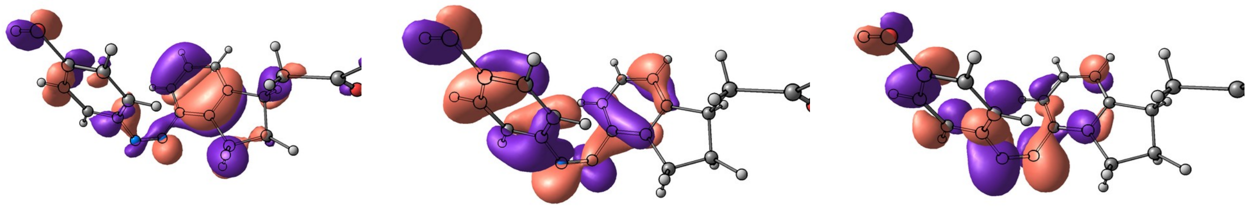

4-aphin cis anti rot 0

ΔE = 3.7655 eV; 329.3 nm

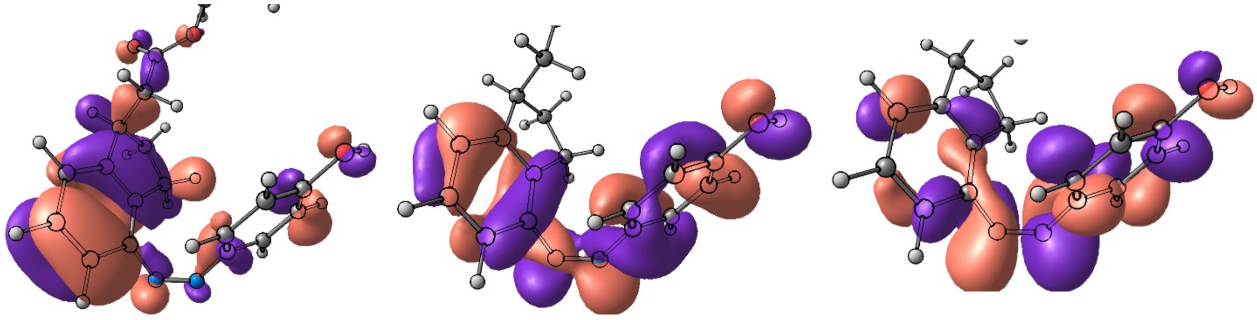

6-aphin trans rot 180

ΔE = 3.5987 eV; 344.5 nm

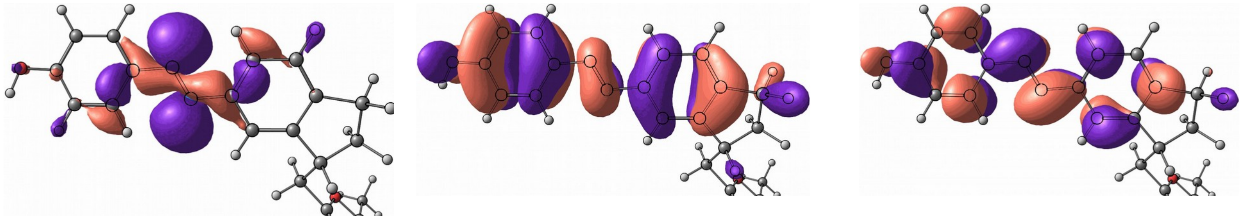

6-aphin trans rot 0

ΔE = 3.5903 eV; 345.3 nm

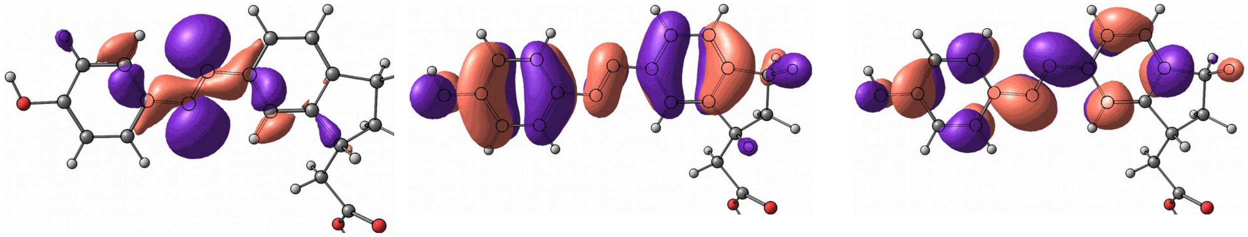

6-aphin cis syn rot 180

ΔE = 3.6270 eV; 341.8 nm

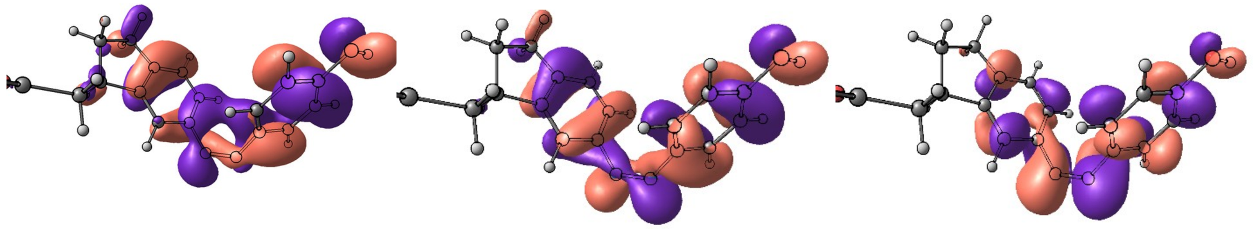

6-aphin cis syn rot 0

ΔE = 3.6115 eV; 343.3 nm

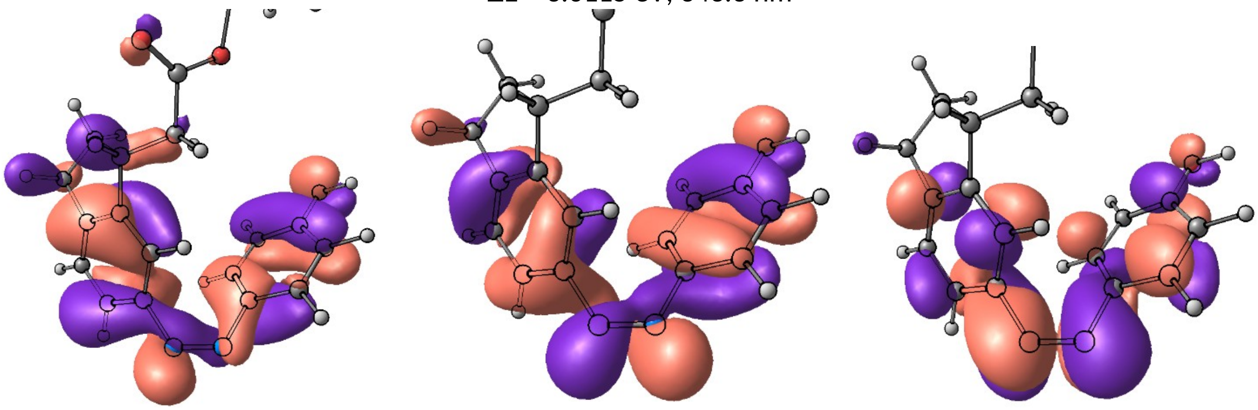

6-aphin cis anti rot 180

ΔE = 3.6153 eV; 342.9 nm

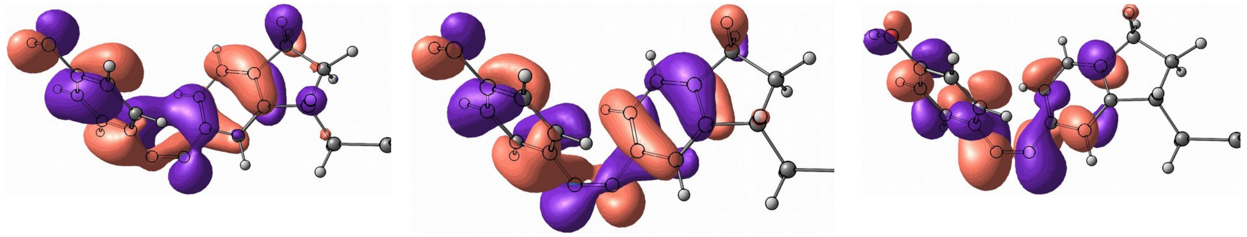

6-aphin cis anti rot 0

ΔE = 3.6034 eV; 344.1 nm

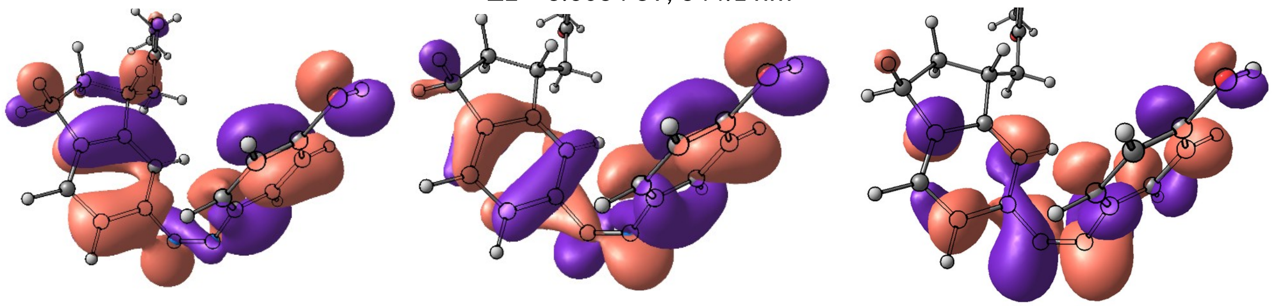

Supplement: Supplementary file 1 [file molecules-27-06716-s001.zip › molecules-1924033-supplementary.pdf]
